# Diagnostic performance of CL Detect rapid-immunochromatographic test for cutaneous leishmaniasis: a systematic review and meta-analysis

**DOI:** 10.1186/s13643-023-02422-y

**Published:** 2023-12-20

**Authors:** Behailu Taye Gebremeskele, Gashaw Adane, Mohammed Adem, Fitsumbrhan Tajebe

**Affiliations:** 1 Department of Medical Laboratory Science, College of Medicine and Health Science, Dilla University, Dilla, Ethiopia; 2 Department of Immunology and Molecular Biology, School of Biomedical and Laboratory Science, College of Medicine and Health Science, University of Gondar, Gondar, Ethiopia

**Keywords:** Cutaneous leishmaniasis, CL Detect rapid test, Diagnosis, Systematic review, Meta-analysis

## Abstract

**Background:**

Sensitive, robust, and fast point-of-care tests are needed for cutaneous leishmaniasis (CL) diagnosis. The recently developed CL Detect rapid test (InBios) for detecting *Leishmania* peroxidoxin antigen has been evaluated in several studies. However, diagnostic performances were controversial. Therefore, this systematic review and meta-analysis aimed to determine the pooled sensitivity and specificity of CL Detect for CL diagnosis.

**Methods:**

PubMed, Scopus, EMBASE, ScienceDirect, and Google Scholar were sources of articles. We included studies reporting the diagnostic accuracy of CL Detect and CL-suspected patients in the English language. The methodological qualities of the included studies were appraised using the quality assessment of diagnostic accuracy studies-2 (QUADAS‐2). Meta-analysis was conducted using Stata 14.2 and R software.

**Results:**

A total of 9 articles were included. The study sample size ranged from 11 to 274. The sensitivities of the individual studies ranged from 23 to 100%, and the specificities ranged from 78 to 100%. Pooled sensitivity and specificity were 68% (95% CI, 41–86%) and 94% (95% CI, 87–97%), respectively. AUC displayed 0.899. Pooled sensitivity was lower (47%, 95% CI, 34–61%) when PCR was used as a reference than microscopy (83%, 95% CI, 39–97%). Pooled sensitivity was lower (48%, 95% CI, 30–67%) for all lesion durations compared to ≤ 4 months (89%, 95% CI, 43–99%).

**Conclusions:**

CL Detect has poor sensitivity and does not meet the minimal sensitivity of 95% of target product profiles designed for CL point-of-care tests. Currently, the CL Detect test looks unsuitable for CL diagnosis, despite its high specificity. Findings are limited by the low number of studies available. Further large-scale studies are recommended.

**Systematic review registration:**

PROSPERO CRD42022323497.

**Supplementary Information:**

The online version contains supplementary material available at 10.1186/s13643-023-02422-y.

## Background

Leishmaniasis is a neglected tropical disease (NTD) caused by various obligate intracellular protozoan parasites of the genus *Leishmania* [1], which are transmitted by the bite of feminine *phlebotomine* sandflies [2]. Cutaneous leishmaniasis (CL) is the most prevalent type of leishmaniasis, manifesting as nodular or ulcerative skin lesions [3]. Beyond the 20 different *Leishmania* species causes CL, the major species are *Leishmania (L.) amazonensis*,* L. mexicana*,* L. Viannia (V.) braziliensis*,* L. (V.) panamensis*, and* L. (V.) guyanensis* in South and Central America (New World), while *L. aethiopica*,* L. tropica*,* L. major*, and* L.* *infantum* in Asia, South Europe, the Middle East, and Africa (Old World) [4–7]. Globally, an estimated 0.7 to 1.2 million CL cases occur annually [2]. Afghanistan, Algeria, Colombia, Brazil, Iran, Syria, Ethiopia, North Sudan, Costa Rica, and Peru are the ten most heavily affected countries, contributing 70 to 75% of the burden [8–10].

Clinically, CL has a spectrum of symptoms such as localized CL (LCL), self-healing nodular or ulcerative lesions at the site of bite; Muco-CL (MCL), destructive nasal, mouth, and throat mucosa; diffuse CL (DCL), multiple non-ulcerative nodules; and disseminated leishmaniasis (DL), multiple papules in two or more non-contiguous areas [11, 12]. These clinical forms are determined by the parasite species and host immunity [13]. Although not fatal, CL can prime severe skin disfiguring and scars, which substantially lead to poor quality of life, societal stigma, and psychiatric problems [14].

There are several diagnostic methods for leishmaniasis, such as parasitological, molecular, and immunological methods [15]. Microscopy, histopathology, and culture are among the parasitological methods [16]. Both culture and histopathology have reduced sensitivity, are time-consuming, and require trained personnel, and culture is costly and hard to make [17]. Microscopy of Giemsa-stained smear taken by skin scraping or needle aspiration is the most commonly used method for CL diagnosis. However, it has poor sensitivity [18, 19] that lies between 15 and 83% depending on the reference test used [15, 20, 21], duration of the lesion [21], parasite load [22], examiner expertise [23], and the prior use of treatments [24]. In addition, sampling causes pain, bleeding, and scars [25].

Compared to microscopy, different molecular methods that have greater accuracy and applicable to minimally invasive samples have evolved for CL diagnosis [4, 26]. Conventional or quantitative polymerase chain reaction (PCR) [27], loop-mediated isothermal AMPlification (LAMP) [28], high-resolution melting (HRM)-PCR [29], and recombinase polymerase assay (RPA) [17] are among them. Despite their superior sensitivity and specificity, molecular methods demand expensive equipment [30], cold-chain-stored reagents [31], special facilities, expertise [32], and DNA/RNA extraction [33]. All these conditions are not accessible in remote settings where most patients are living [18, 30].

*Leishmania* diagnosis was more frequently performed using immunoglobulin-based immunological techniques such as western blotting, ELISAs, and indirect immuno-fluorescence assays (IFAT) [34]. However, due to their lower sensitivity and the fact that CL patients do not secrete enough antibodies to fight parasites, these tests were not frequently employed to diagnose CL [15]. Although highly sensitive, antigen-based Leishmania intradermal skin tests (LST) and Montenegro skin tests (MST) could not distinguish between present and past infections [5, 15]. Antigen-based rapid point-of-care (POC) tests that are extremely sensitive, trustworthy, quick, and robust are thus essential for decentralizing CL diagnosis at primary healthcare facilities, especially in countries with limited resources [35, 36]. Using polyclonal antibodies and dental broach sampling, the CL Detect rapid test (InBios International Inc., Seattle, USA) targets the *Leishmania* peroxidoxin antigen [37].

A CL Detect rapid test has been evaluated in several CL diagnostic accuracy studies across the world. However, diagnostic performances were inconsistent and controversial. Moreover, there was no previously done systematic review and meta-analysis on the accuracy of this test across the world. Therefore, this systematic review and meta-analysis was designed to estimate the pooled sensitivity and specificity of the CL Detect rapid test across the world using the available evidence. If the CL Detect rapid ICT works well, this could revolutionize CL diagnosis.

## Methods

### Literature review protocol preparation

This systematic review and meta-analysis were registered on the International Prospective Register of Systematic Reviews (CRD42022323497). In addition, this study was carried out following the guidelines of the Preferred Reporting Items for Systematic Review and Meta-Analysis (PRISMA) [38].

### Information sources and search strategy

Data were gathered from PubMed, Scopus, EMBASE, ScienceDirect, and Google Scholar by searches using the key terms (“cutaneous leishmaniasis” OR “Leishmaniasis, American” OR “Leishmaniasis, New World” OR Leishmaniasis, Old World” OR “Oriental Sore” OR “American tegumentary leishmaniasis”) AND (“CL Detect rapid test” OR “CL Detect rapid immunochromatographic diagnostic test” OR “antigen based point of care test”)). An additional filter in English language was used. Other publications were recognized from references cited in important articles and manually hand-searched to identify further pertinent studies (see Additional file 1: Text S1).

### Eligibility criteria 

Studies were included if observational or cross-sectional diagnostic accuracy was published in English language, CL suspected patients involved, CL Detect test and microscopy or molecular tests were performed, and numbers of true positive (TP), false positive (FP), true negative (TN), and false negative (FN) were directly or indirectly available. We excluded case reports, review articles, meta-analysis articles, studies with incomplete data, and duplicates.

### Study selection 

The retrieved articles were imported to EndNote X8, and duplicate articles were removed. Then, articles were screened by their titles, abstracts, and full text according to the eligibility criteria by two reviewers (BT and FT) independently. Since there were no disagreements, no article is resolved with a third reviewer or by consensus.

### Data extraction

Data extraction was performed by reviewers. Variables extracted were the first author’s name, year of publication, country, geographic region, continent, study design, duration of lesion (DL), index test sampling method, study population, sample size, cases, reference test, true positive (TP), false positive (FP), true negative (TN), and false negative (FN) of CL Detect rapid test.

### Quality assessment

Two reviewers (BT and FT) assessed the risks of bias and applicability concerns using the quality assessment of diagnostic accuracy studies 2 (QUADAS-2) tool [39]. Evaluation results were displayed in graphs using Review Manager 5.4 software.

### Statistical analysis

Data were extracted in Excel and then exported to Stata version 14.2 for analysis. A random-effects model was employed using the Metadta package for meta-analysis. The degree of heterogeneity was quantified using *I*-square (*I*^2^) statistics by Zhou and Dendukuri [40] in Stata and R software. *I*^2^ values above 25%, 50%, and 75% were assumed to be low, medium, and high heterogeneity, respectively. To resolve high heterogeneity, sub-group analysis (by reference, lesion duration, and continent) followed by sensitivity analyses (using the MathiasHarrer/dmetar package in R) were performed. Deeks’ funnel plot and Egger’s statistics were done to detect publication bias. A *p* value of ≤ 0.05 in Egger’s test was considered evidence of statistically significant publication bias [41].

## Results

### Literature search

**Fig. 1:**
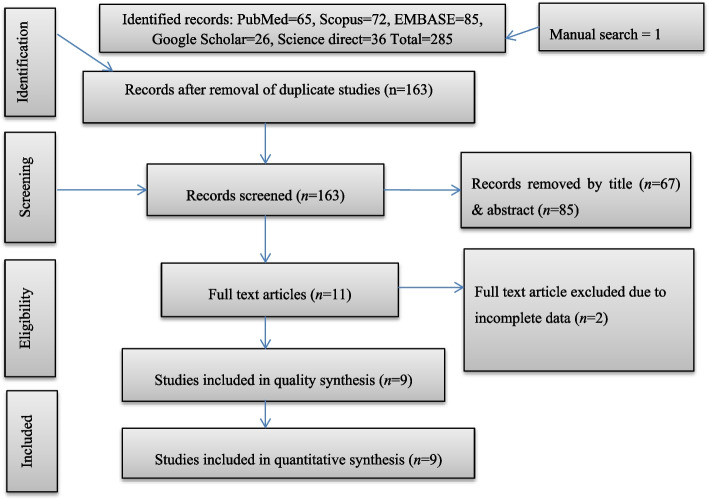
Flow chart of the study selection process. *n* number of articles

A total of 285 studies were retrieved from databases and manual searches. After removing duplicates, 163 articles were screened by title and abstract and 11 by full-text reading. Articles were excluded at the title/abstract screening stage due to not being in the field of interest (CL), diagnostic tests different from CL Detect, and non-full text. Two studies were excluded during the full-text screening phase due to incomplete data [42, 43]. Finally, nine studies were included for the qualitative and quantitative analysis (Fig. 1).

### Description of included studies

In this systematic review and meta-analysis, nine articles were included, and five of them had two diagnostic test results per patient due to two kinds of reference tests or sampling applied, resulting in 14 total observations (datasets) (Additional file: Table S1). The majority [7] of studies evaluated the CL Detect performance in the Old World (Morocco, India, Sri Lanka, Ethiopia, Afghanistan, Tunisia, and Iran), and two studies evaluated its performance in the New World (Suriname and Peru). This rapid test accuracy was evaluated using combined microscopy and/or PCR as a reference test in 3 studies, microscopy in 2 studies, PCR only in a single study, and microscopy and PCR distinctly in 2 studies. The sample size of the included studies ranged from 11 to 274. In total, 1229 individuals and 1713 test results for the diagnosis of CL were included in this review. Of the 1713 test results; 801, 657, and 255 were assessed under microscopy and/or PCR, microscopy, and PCR reference tests, respectively. Four studies evaluated the CL Detect performance in patients with less than or equal to a 4-month lesion duration (referred to us, LFM), whereas the other 4 studies assessed patients with all lesion duration (referred to us, PAD). A single study did not mention the age of the lesion (unknown). All the studies applied dental broach sampling, and two studies used additional skin-slit sampling for the CL-Detect test.

### Methodological qualities of included studies using QUADAS-2

**Fig. 2:**
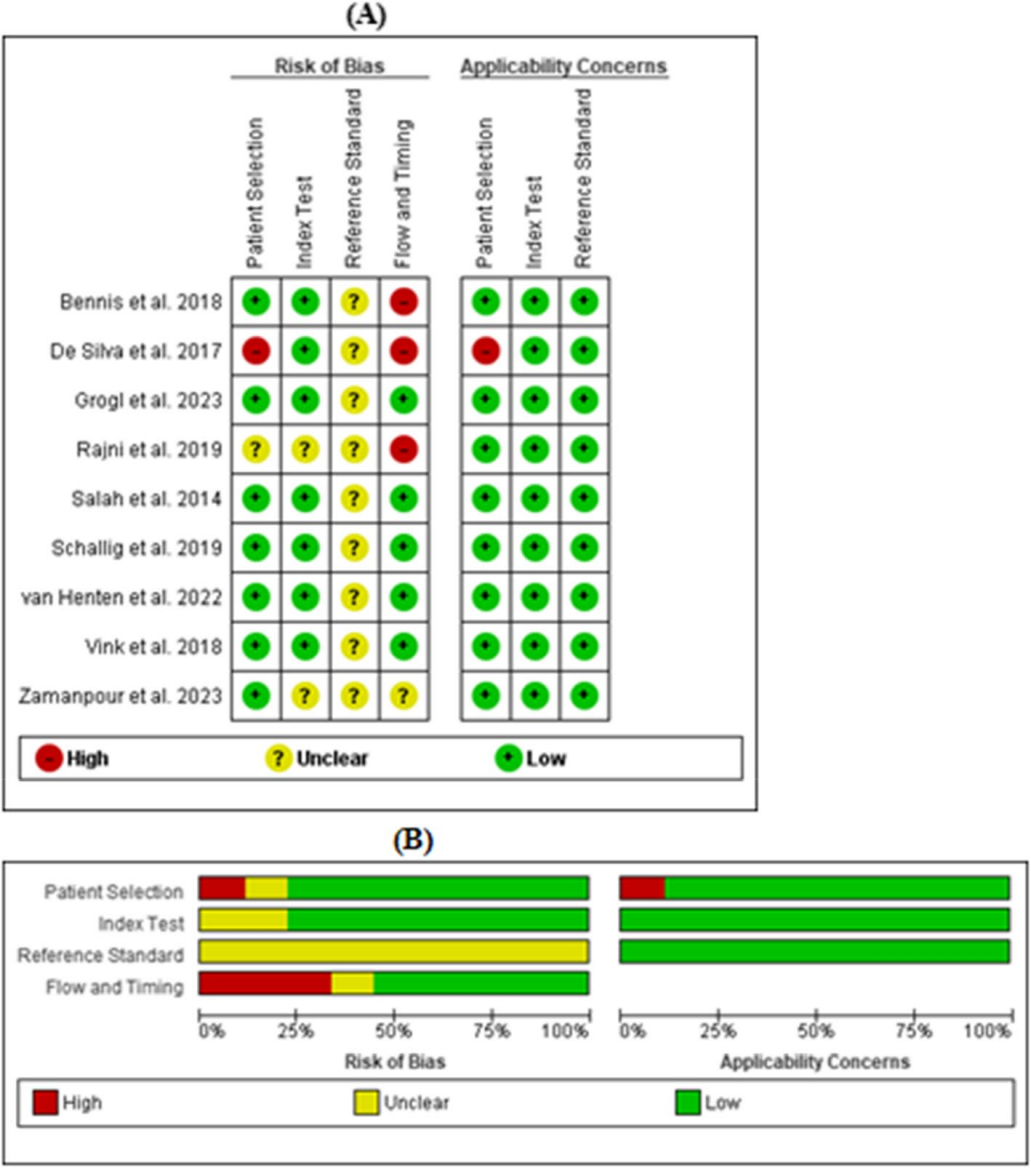
**A** Risk of bias and applicability concerns summary. **B** Percentages across included studies using the QUADAS-2 tool

The methodological qualities of all included studies regarding the applicability concern were judged as low in all three domains (patient selection, index test, and reference standard), except for a single study [44], which was classified as high in the patient selection domain due to some participants (controls) mismatching the review question (Fig. 2). The majority of the studies were rated as having a low risk of bias in both patient selection and index test domains. Five studies have a low risk of bias for domain flow and timing, with three having a high and one having an unclear risk of bias regarding this domain. Because there was no gold standard [45] or highly sensitive (100%) and specific test to correctly classify CL, all studies were judged unclear for the reference standard domain.

### Pooled sensitivity and specificity of CL Detect rapid test for CL diagnosis

**Fig. 3:**
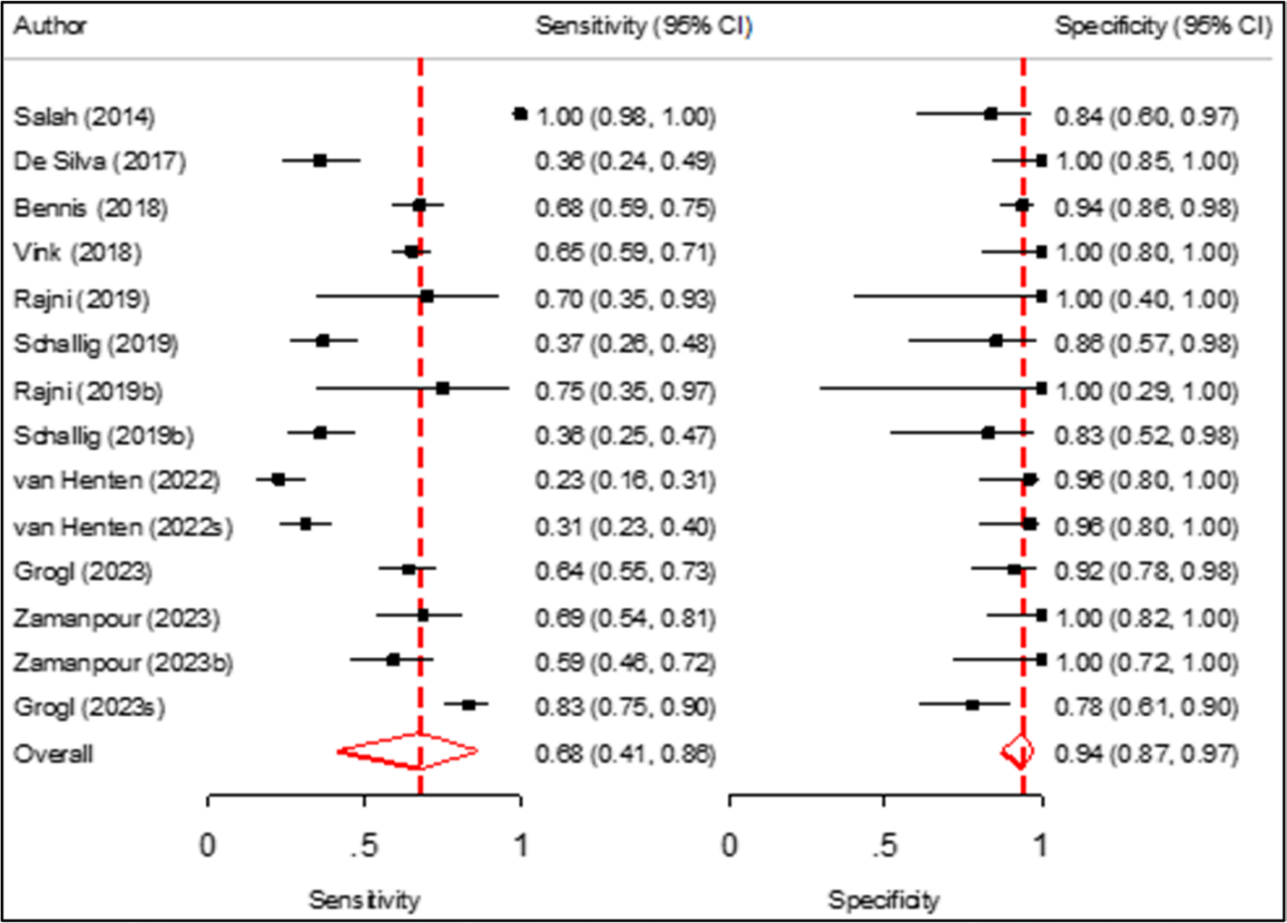
Forest plot for pooled sensitivity and specificity of CL Detect rapid test for CL diagnosis. Value and pooled estimate (last rows per sensitivity and specificity analysis, red diamond)

The sensitivities of the individual studies ranged from 23 to 100%, and the specificities ranged from 78 to 100% (Fig. 3). The pooled specificity of the CL Detect rapid test for CL diagnosis was 94% (95% CI 87–97%, *p* value 0.001), and the pooled sensitivity demonstrated 68% (95% CI, 41–86%, *p* value 0.191). The heterogeneity for sensitivity was (Tau-squared = 2.73, *I*^2^ = 85%) and (Tau-squared = 0.54, *I*^2^ = 15%) for specificity.

### Diagnostic accuracy of CL Detect rapid test for CL diagnosis using SROC curve

**Fig. 4:**
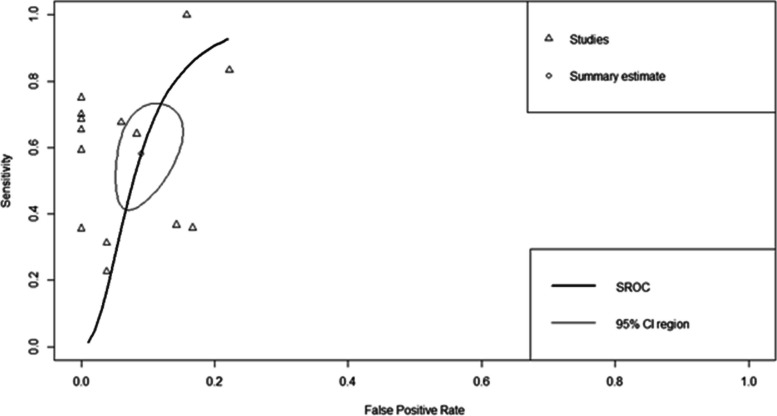
SROC curve for CL Detect rapid test for CL diagnosis. SROC summary receiver operating characteristic. Arrows represent the single study data, and circles indicate summary estimates with 95% CI

The summary diagnostic accuracy of the CL Detect rapid test for CL diagnosis was presented by the SROC plot (Fig. 4). The observed data (arrows) were extremely scattered around the summary point estimate (circle) that might be due to differences in the diagnostic accuracy of the test across different populations. The AUC value displayed 0.899, implying that CL Detect had good in overall diagnostic accuracy.

### Subgroup analysis for pooled sensitivity of CL Detect rapid test for CL diagnosis

First, the Spearman correlation coefficient was tested to assess whether the threshold effect was a cause of high heterogeneity. A correlation coefficient of − 0.082 was obtained, indicating heterogeneity was not due to the threshold effect.

Next, a subgroup analysis was performed (Table 1). The sensitivity in the studies that used microscopy as a reference test was higher (83%, 95% CI, 39–97%) than in the studies that used the reference standard of combined microscopy or/and PCR (53%, 95% CI, 31–74%) and PCR alone (47%, 95% CI, 34–61%). The values of *I*^2^ were 93%, 72%, and 51% for microscopy or/and PCR, microscopy, and PCR reference test subgroups, respectively. Similarly, studies that evaluated CL Detect on LFM patients displayed higher pooled sensitivity (89%, 95% CI, 43–99%) compared to studies that assessed its performance among PAD (48%, 95% CI, 30–67%). The value of *I*^2^ was 85% for LFM and 61% for the PAD subgroup. Additionally, pooled sensitivity was found good (90%) with a wider CI (11–100%) for CL patients from Africa, but it was lower for CL patients from Asia (59%, 95% CI, 45–71%) and America (56%, 95% CI, 29–79%). For subgroups Africa, Asia, and America, *I*^2^ was 20%, 43%, and 86%, respectively.

**Table 1:** Subgroup analysis for pooled sensitivity of CL Detect rapid test for CL

Subgroup by		No. of studies (no. of observations)	*I*^2^%	*p* value	Pooled sensitivity (95% CI)
Reference test	Microscopy/PCR	3 (4)	93	0.789	53 (31–74)
	Microscopy	5 (6)	72	0.126	83 (39–97)
	PCR	4 (4)	51	0.678	47 (34–61)
DL	LFM	4 (5)	85	0.085	89 (43–99)
	PAD	4 (7)	61	0.084	48 (30–67)
Continent	Africa	3 (4)	20	0.312	90 (11–100)
	Asia	4 (6)	43	0.201	59 (45–71)
	America	2 (4)	86	0.674	56 (29–79)

*I*^2^ heterogeneity, *DL* duration of lesion, *LFM* less than 4 months of DL, *PAD* patients with all DL, *CI* confidence interval

### Sensitivity analysis

**Fig. 5:**
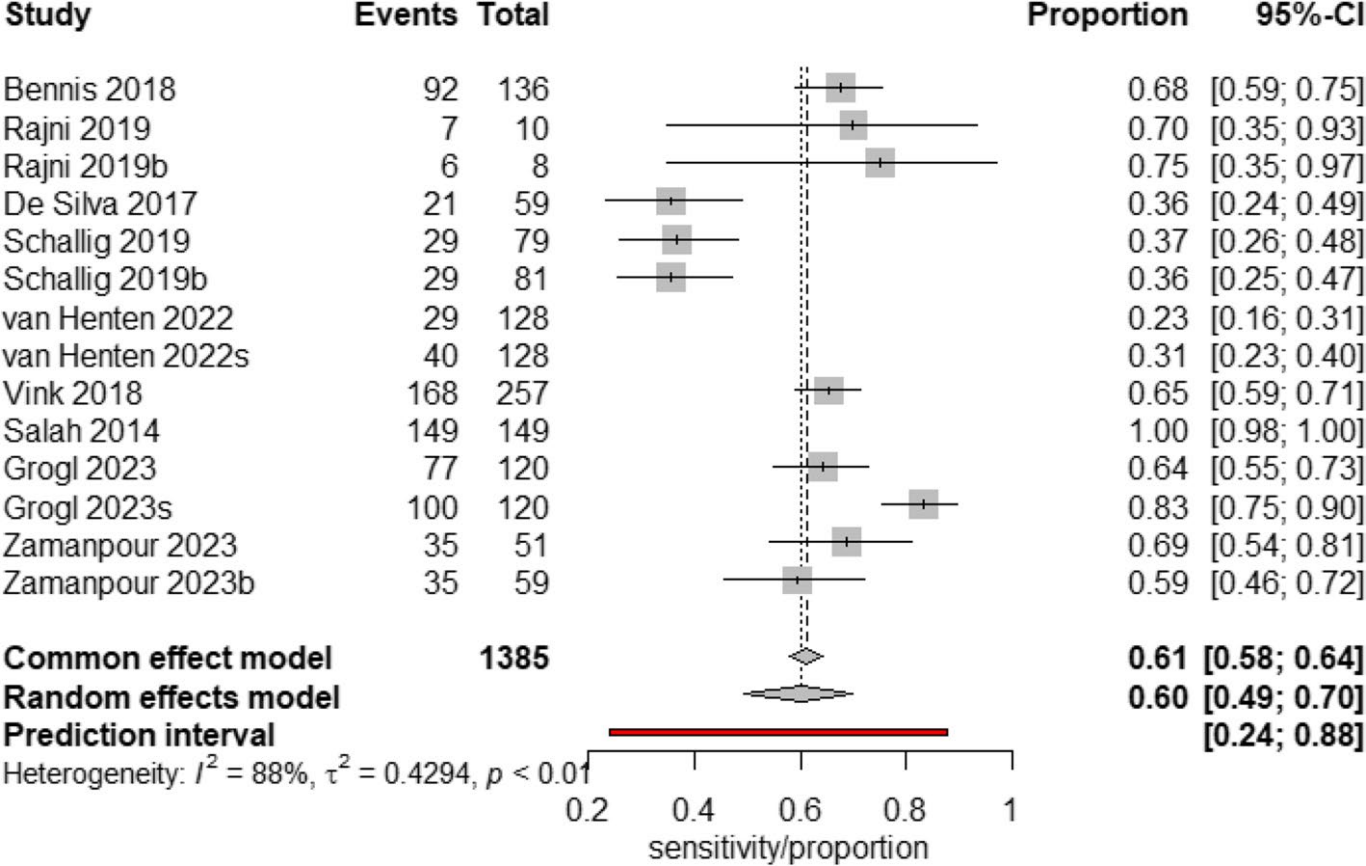
Forest plot for pooled sensitivity of CL Detect test for CL diagnosis after removing outliers. Value and pooled estimate (last row per sensitivity analysis, black diamond). *I*^2^ heterogeneity

To further explore the heterogeneity of pooled sensitivity, a sensitivity analysis was conducted. The studies “van Henten et al.” and “Salah et al.” were identified as outliers using a random-effects model. The sensitivity of the CL Detect rapid test with outliers removed was 60% (95% CI, 49–70%, *p* value < 0.01) (Fig. 5). The heterogeneity was (Tau-squared = 0.42, *I*^2^ = 88%).

An influential analysis was conducted to assess the study. The pooled sensitivity ranges from 54% (95% CI, 43–66%) to 66% (95% CI, 46–81%) via omitting studies by Salah et al. [46] and van Henten et al. [47], respectively. The influence of each study on the diagnostic sorted by heterogeneity contribution showed the study “van Henten et al.” contributed both the high heterogeneity (43.7) and effect size (36.3). Finally, the leave-one-out analysis shows the highest (*I*^2^ = 92.7%) to the lowest (*I*^2^ = 88.8%) through omitting every study, indicating no significant reduction of heterogeneity (see Additional file: Table S2).

### Publication bias

The included studies were assessed for potential publication bias visually by Deeks’ funnel plot and Egger’s statistics. In this systematic review and meta-analysis, the Deeks’ funnel plots of the included studies were almost symmetric and the Egger-weighted regression statistics had a nonsignificant value (*p* value = 0.79), indicating that there was no potential publication bias (see Additional file 4: Figure 1).

## Discussion 

The world is pressing for antigen-based, sensitive, reliable, and easy-to-use POC tests for prompt and decentralized diagnosis and control of CL, particularly in resource-limited areas [35]. The CL Detect rapid test is designed as a rapid diagnostic tool for *Leishmania* antigen detection and can be used in primary healthcare facilities, which could improve the diagnosis of CL. To our knowledge, this is the first systematic review with meta-analysis to analyze and summarize the diagnostic accuracy of the CL Detect rapid test for CL, using all relevant studies from the available literature.

In this meta-analysis, the pooled sensitivity of the CL Detect rapid test for CL diagnosis was found inferior and unmet the minimal sensitivity of 95% of target product profiles (TPPs) designed for antigen-based POC tests for CL [35]. The pooled specificity of the CL Detect was high, and it fulfills TPPs criteria for CL diagnostic tests, which request at least 90% specificity to avoid related non-CL dermal diseases [35]. The pooled SROC showed an AUC of 0.899 for this rapid test, reflecting a good distinguishing capacity of CL cases from non-cases. However, its AUC does not reach 0.97, and the summary estimate was not closer to the top left corner to claim excellent diagnostic accuracy [48, 49], which represents high sensitivity and specificity.

Due to the significant heterogeneity in pooled sensitivity, we conducted a subgroup analysis. Although CL Detect pooled sensitivity becomes high (83%) when microscopy is used as a reference test, it declines to 53% and 47% when PCR alone or combined with a microscope as a reference test, respectively. This is considerably due to the high sensitivity of PCR in contrast to a microscope [20, 42, 50], which is not sufficiently sensitive by itself to be used as a reference standard. CL Detect was initially developed to detect CL in less than 4-month-old lesions [37], and we found high (89%) pooled sensitivity for these patients. However, this sensitivity potential was reduced by 40% for patients of all lesion durations. This is presumably due to the fact that the early lesion has a high parasite load rather than aged [51, 52]. While this rapid test revealed high sensitivity in CL patients in Africa, it performed moderately well sensitivity for patients in Asia and America. This might be explained by the variation in expression level or alteration of target antigens among species. New World species and *L.* *donovani* predominately cause CL in Sri Lanka, and some parts of India (Asia) were indicated to express low peroxidoxin targeted by the CL Detect [19, 44]. Moreover, the CL Detect limit of detection was above five times for New World species and almost twice for *L.* *donovani* compared to *L.* *tropica* and *L. major* [37]. All pooled sensitivities, including those obtained as high in some subgroups, cannot address a sensitivity of at least 95% in parasitologically confirmed patients [35].

In this meta-analysis, we also performed sensitivity analysis to further demonstrate the potential heterogeneity of pooled sensitivity, which showed a statistically significant (*p* value < 0.05) estimate (60% pooled sensitivity). This poor sensitivity might be due to inferior concentration or mutant peroxidoxin formation, as presumed for *L.* *donovani* and New World species [19, 44], which makes the target undetectable or missed by the test. Also, the parasite number required to become test-positive highly differs among as well as within species [37], which makes the test less sensitive in low parasite specimens when high concentrations are expected. Primarily, *Leishmania* parasites produce peroxidoxin to evade host killing mechanism inside microphages through reactive oxygen and nitrogen species [53, 54]. Hence, low peroxidoxin implies more susceptibility of *Leishmania* parasites to host immunity on the one hand and parasite inadequate detoxification of reactive oxygen and nitrogen species on the other hand [55]. Furthermore, CL Detect poor sensitivity might be associated with highly sensitive PCR [20, 42, 50] employed as a reference test in most studies of this review rather than less sensitive microscopy, which could inflate CL Detect sensitivity.

Overall, this systematic review and meta-analysis showed the current CL Detect rapid test (InBios International Inc., Seattle, USA) is not suitable for CL diagnosis because of its limited sensitivity. This review clearly demonstrated that using this rapid test only on very young lesions as per its intended purpose also unmet the minimum sensitivity of 95% requirements for a PoC test for CL developed by the Foundation for Innovative New Diagnostics (FIND) [35], despite the fact that it has good specificity and is user-friendly. Deploying such an inadequate sensitivity test can hamper early diagnosis, effective treatment, and control of CL, in addition to underestimating the infection rate. Thus, further research is needed to develop simple PoC tests with high sensitivity and perform equally well across CL species, as well as meet the specified requirements of TTPs to allow decentralization in the diagnosis and care of CL.

The strengths of this systematic review and meta-analysis are the employment of different searching strategies, critical appraisal of the methodological quality of included studies using the QUADAS-2 tool, application of the PRISMA 2020 guideline, and conducting sensitivity analysis. However, this review has several limitations. A major limitation is the presence of heterogeneity and a lack of statistical significance (*p* value < 0.05) in most results. Subsequently, the results have to be interpreted with caution. High heterogeneity was not due to the threshold effect, so it might be due to several factors like prevalence and demographic factors of the sample population [56, 57]. Additionally, the evaluation result of QUADAS-2 shows some of the studies have a high risk of bias for the flow and timing domain, and many studies have an unclear risk of bias for the reference standard domain because there is no gold standard test for CL. Lastly, considering eight patients who have above 4 months and unknown lesion duration as ≤ 4 months in Vink et al.’s [18] study, since the rest (266/274) have ≤ 4 months and are not significantly affected as we believe.

## Conclusions

CL Detect rapid test sensitivity was poor compared to the least expected (> 95% sensitivity) set by WHO as well as FIND for antigen-based tests for CL, despite its high specificity. Currently, the CL Detect test looks unsuitable for CL diagnosis, although the need for rapid tests in CL endemic settings remains high. These findings are limited by a low number of studies available; hence, further large-scale studies evaluating the CL Detect test in different endemic areas with various factors (like *Leishmania* species, peroxidoxin expression level, CL type, host immune status, and multiple sampling) are recommended to gain further insights.

### Supplementary Information

**Additional file 1: Text S1.** Search strategy. Shows the specific searches conducted in the databases using the key terms to identify studies included in the review.

** Additional file 2:** **Table S1.**Summery of included studies in this systematic review and meta-analysis.

** Additional file 3:** **Table S2.** Leave-one-out analysis and diagnostic influence effect size for sensitivity of CL Detect test sorted by heterogeneity contribution.

** Additional file 4:** **Figure S1. **Deeks’ funnel plot of CL Detect rapid test for CL diagnosis.

## Data Availability

Data we used for this study are available within the review and its additional files.

## Abbreviations

CL: Cutaneous leishmaniasis
CI: Confidence intervals
DCL: Diffuse CL
DL: Duration of lesion
FIND: Foundation for Innovative New Diagnostics
FN: False negative
FP: False positive
ICT: Immuno-chromatographic test
LCL: Localized CL
MCL: Muco-CL
PCR: Polymerase chain reaction
POC: Point-of-care
PRISMA: Preferred Reporting Items for Systematic Review and Meta-Analysis
QUADAS-2: Quality assessment of diagnostic accuracy studies-2
SROC: Summary receiver operating characteristic
TP: True positive
TN: True negative
TPPs: Target product profiles
WHO: World Health Organization
