# Supplementary material for: Diagnostic performance of CL Detect rapid-immunochromatographic test for cutaneous leishmaniasis: a systematic review and meta-analysis
Source: Syst Rev. 2023 Dec 20;12:240. doi: 10.1186/s13643-023-02422-y (PMC10731771; doi:10.1186/s13643-023-02422-y)
Supplement: Supplementary file 1 — Additional file 1: Text S1. Search strategy. Shows the specific searches conducted in the databases using the key terms to identify studies included in the review. [file 13643_2023_2422_MOESM1_ESM.docx]

**Additional file 1: Text S1.** Search strategy**.** Shows the specific searches conducted in the databases using the key terms to identify studies included in the review.

The searching terms used in PubMed were (Cutaneous leishmaniasis[Title/Abstract] OR Leishmaniasis, American[Title/Abstract] OR Leishmaniasis, New World[Title/Abstract] OR Leishmaniasis, Old World[Title/Abstract] OR Oriental Sore[Title/Abstract] OR American tegumentary leishmaniasis[Title/Abstract]) AND (CL Detect rapid test[Title/Abstract] OR CL Detect rapid immunochromatographic diagnostic test[Title/Abstract] OR antigen based point of care test[Title/Abstract]).

The searching terms used in Scopus were (Title-Abs-Key (“Cutaneous leishmaniasis” OR “Leishmaniasis, American” OR “Leishmaniasis, New World” OR Leishmaniasis, Old World” OR "Oriental Sore" OR "American tegumentary leishmaniasis")) AND Title-Abs-Key ("CL Detect rapid test" OR "CL Detect rapid immunochromatographic diagnostic test" OR "antigen based point of care test"))).

The searching terms used in EMBASE were (Cutaneous leishmaniasis [Title/Abstract] OR Leishmaniasis, American [Title/Abstract] OR Leishmaniasis, New World [Title/Abstract] OR Leishmaniasis, Old World [Title/Abstract] OR Oriental Sore [Title/Abstract] OR American tegumentary leishmaniasis[Title/Abstract]) AND (CL Detect rapid test [Title/Abstract] OR CL Detect rapid immunochromatographic diagnostic test [Title/Abstract] OR antigen based point of care test [Title/Abstract]).

The searching terms used in Science direct were (Cutaneous leishmaniasis OR Leishmaniasis, American OR Leishmaniasis, New World OR Leishmaniasis, Old World OR Oriental Sore OR American tegumentary leishmaniasis) AND (CL Detect rapid test OR CL Detect rapid immunochromatographic diagnostic test OR antigen based point of care test).

The searching terms used in Google scholar were (With all of the words: "Cutaneous leishmaniasis" OR "Leishmaniasis, American" OR "Leishmaniasis, New World" OR "Leishmaniasis, Old World" OR "Oriental Sore" OR "American tegumentary leishmaniasis" AND "CL Detect rapid test" OR "CL Detect rapid immunochromatographic diagnostic test" OR "antigen based point of care test"), (With the exact phrase: "Cutaneous leishmaniasis" AND "CL Detect rapid test") and (With at least one of the words: CL Detect rapid test). Additional filter such as language (English) was used. Furthermore, other publications were recognized from references cited in important articles and manually hand-searched to identify further pertinent studies.
