# Supplementary material for: Diagnostic performance of CL Detect rapid-immunochromatographic test for cutaneous leishmaniasis: a systematic review and meta-analysis
Source: Syst Rev. 2023 Dec 20;12:240. doi: 10.1186/s13643-023-02422-y (PMC10731771; doi:10.1186/s13643-023-02422-y)
Supplement: Supplementary file 2 — Additional file 2: Table S1.Summery of included studies in this systematic review and meta-analysis. [file 13643_2023_2422_MOESM2_ESM.docx]

Additional file 2: Table S1: Summery of included studies in this systematic review and meta-analysis.

| Author | Year | Country | Region | Study design | Pop | n | Case | TP | FP | FN | TN | Reference | DL | Sampling |
| --- | --- | --- | --- | --- | --- | --- | --- | --- | --- | --- | --- | --- | --- | --- |
| Salah et al. (44) | 2014 | Tunisia | Old | cross-sectional | CL | 168 | 149 | 149 | 3 | 0 | 16 | Microscopy | LFM | DB |
| De Silva et al. (45) | 2017 | Sri Lanka | Old | case-control | CL | 81 | 59 | 21 | 0 | 38 | 22 | PCR | PAD | DB |
| Bennis et al. (46) | 2018 | Morocco | Old | cross-sectional | CL | 219 | 136 | 92 | 5 | 44 | 78 | Microscopy/PCR | LFM | DB |
| Vink et al. (47) | 2018 | Afghanistan | Old | cross-sectional | CL | 274 | 257 | 168 | 0 | 89 | 17 | Microscopy/PCR | LFM | DB |
| Rajni et al. (48) | 2019 | India | Old | cross-sectional | CL | 14 | 10 | 7 | 0 | 3 | 4 | Microscopy | PAD | DB |
| Rajni et al. (48) | 2019b | India | Old | cross-sectional | CL | 11 | 8 | 6 | 0 | 2 | 3 | PCR | PAD | DB |
| Schallig et al. (49) | 2019 | Suriname | New | cross-sectional | CL | 93 | 79 | 29 | 2 | 50 | 12 | Microscopy | UN | DB |
| Schallig et al. (49) | 2019b | Suriname | New | cross-sectional | CL | 93 | 81 | 29 | 2 | 52 | 10 | PCR | UN | DB |
| van Henten et al. (50) | 2022 | Ethiopia | Old | cross-sectional | CL | 154 | 128 | 29 | 1 | 99 | 25 | Microscopy/PCR | PAD | DB |
| van Henten et al. (50) | 2022s | Ethiopia | Old | cross-sectional | CL | 154 | 128 | 40 | 1 | 88 | 25 | Microscopy/PCR | PAD | SS |
| Grogl et al. (51) | 2023 | Peru | New | Cross-sectional | CL | 156 | 120 | 77 | 3 | 43 | 33 | Microscopy | LFM | DB |
| Grogl et al. (51) | 2023s | Peru | New | Cross-sectional | CL | 156 | 120 | 100 | 8 | 20 | 28 | Microscopy | LFM | SS |
| Zamanpour et al. (52) | 2023 | Iran | Old | Cross-sectional | CL | 70 | 51 | 35 | 0 | 16 | 19 | Microscopy | PAD | DB |
| Zamanpour et al. (52) | 2023b | Iran | Old | Cross-sectional | CL | 70 | 59 | 35 | 0 | 24 | 11 | PCR | PAD | DB |

CL: cutaneous leishmaniasis, n: sample size, pop: population, TP: true positive, FP: false positive, TN: true negative, FN: false negative, DL: duration of lesion, LFM: less than or equal to four month, PAD: patients with all lesion duration, UN: unknown, DB: dental broach, and SS: skin-slit.
