# Supplementary material for: Diagnostic performance of CL Detect rapid-immunochromatographic test for cutaneous leishmaniasis: a systematic review and meta-analysis
Source: Syst Rev. 2023 Dec 20;12:240. doi: 10.1186/s13643-023-02422-y (PMC10731771; doi:10.1186/s13643-023-02422-y)
Supplement: Supplementary file 3 — Additional file 3: Table S2. Leave-one-out analysis and diagnostic influence effect size for sensitivity of CL Detect test sorted by heterogeneity contribution. [file 13643_2023_2422_MOESM3_ESM.docx]

**Additional file 3: Table S2: Leave-one-out analysis and diagnostic influence effect size for sensitivity of CL Detect test sorted by heterogeneity contribution**

|  | Pooled sensitivity (95% CI) | I^2^ (%) | Heterogeneity contribution | Influence effect size |
| --- | --- | --- | --- | --- |
| Omitting van Henten 2022 | 66 (48-81) | 89.8 | 43.7 | 36.3 |
| Omitting Grogl 2023s | 61 (41-78) | 90.6 | 34.6 | 3.17 |
| Omitting van Henten 2022s | 66 (46-81) | 91.3 | 25.1 | 29.2 |
| Omitting Salah 2014 | 54 (43-66) | 92.7 | 15.2 | 0.01 |
| Omitting Vink 2018 | 63 (43-80) | 91.9 | 12.7 | 5.10 |
| Omitting Schallig 2019b | 65 (45-81) | 92.2 | 10.5 | 20.4 |
| Omitting Bennis 2018 | 63 (42-80) | 92.2 | 9.67 | 7.49 |
| Omitting Schallig 2019 | 65 (45-81) | 92.3 | 9.32 | 19.8 |
| Omitting De Silva 2017 | 65 (45-81) | 92.4 | 7.83 | 18.2 |
| Omitting Grogl 2023 | 63 (43-80) | 92.5 | 4.75 | 9.46 |
| Omitting Zamanpour 2023 | 62 (42-79) | 92.5 | 4.15 | 10.5 |
| Omitting Rajni 2019b | 62 (42-79) | 92.7 | 1.30 | 12.3 |
| Omitting Rajni 2019 | 63 (43-79) | 92.7 | 0.97 | 12.3 |
| Omitting Zamanpour 2023b | 64 (43-80) | 92.7 | 0.62 | 12.2 |
