# Supplementary figures and images for: Diagnostic performance of CL Detect rapid-immunochromatographic test for cutaneous leishmaniasis: a systematic review and meta-analysis
Source: Syst Rev. 2023 Dec 20;12:240. doi: 10.1186/s13643-023-02422-y (PMC10731771; doi:10.1186/s13643-023-02422-y)

**Additional file 4: Figure S1. Deeks’ funnel plot of CL Detect rapid test for CL diagnosis.**


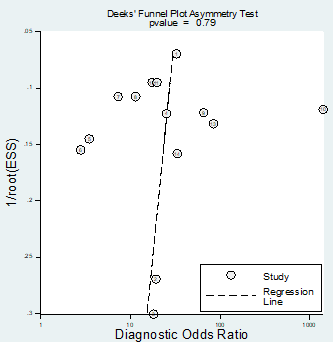

Supplement: Supplementary file 4 — Additional file 4: Figure S1. Deeks’ funnel plot of CL Detect rapid test for CL diagnosis. [file 13643_2023_2422_MOESM4_ESM.docx]
